# Abdominal Ectopic Pregnancy and Impaired Postnatal Mammary Gland Development, Consistent With Physiologic Agalactia, in a Wild European Rabbit, *Oryctolagus cuniculus*

**DOI:** 10.3389/fvets.2019.00254

**Published:** 2019-08-07

**Authors:** Katherine Hughes

**Affiliations:** Department of Veterinary Medicine, University of Cambridge, Cambridge, United Kingdom

**Keywords:** abdominal pregnancy, ectopic pregnancy, extrauterine pregnancy, lithopedion, mammary gland, *Oryctolagus cuniculus*, pregnancy, rabbit

## Abstract

A wild European rabbit, *Oryctolagus cuniculus*, was diagnosed with an abdominal pregnancy due to the presence of a single abdominal lithopedion attached by a thin fibrovascular stalk to the left uterine horn, which was distorted by the tension of the adhesion. Evidence of mineralized remnants, chronic inflammation, and fibrosis in the left uterine endometrium and myometrium suggested that the lithopedion had arisen as a secondary abdominal pregnancy. The right uterine horn contained two macroscopically normal fetuses. The mammary gland exhibited notably retarded development in relation to the size of the fetuses. Histologically, mammary alveoli lacked evidence of intraluminal secretory product, and ducts lacked prominence and contained clusters of small numbers of macrophages. The doe also exhibited mild granulomatous and heterophilic pneumonia with rare intralesional adiaspores, suggesting infection with *Emmonsia* spp. as an incidental finding. This case documents secondary abdominal pregnancy in a wild lagomorph not subjected to artificial insemination procedures suggested to increase the occurrence of this condition in farmed rabbits. An abdominal pregnancy is one of a number of factors that should be considered as a potential factor in the etiology of impaired postnatal mammary development or reduced milk yield in a breeding doe, although no causative association is demonstrated in this case. Abdominal ectopic pregnancy is one possible differential diagnosis in the investigation of the presence of a palpable abdominal mass or masses in *O. cuniculus*.

## Background

Extrauterine pregnancy, often used synonymously with the term “ectopic pregnancy,” describes a pregnancy occurring outwith the uterus (1). Four subtypes are recognized (Table 1) but the most common subtype arising in animals, particularly in species other than non-human primates, is abdominal pregnancy. Primary abdominal pregnancy arises when the conceptus implants within the abdomen due to escape from the fallopian tube, for example following retrograde movement through the fimbria. By contrast, secondary abdominal pregnancy occurs following uterine or tubal trauma that results in abdominal implantation.

**Table 1:** Tabular representation of subtypes of extrauterine pregnancy, as recognized in human medicine, with selected case examples from humans and animals.

**Extrauterine pregnancy** Often used synonymously with the term “ectopic pregnancy” to describe a pregnancy occurring outwith the uterus (1).				
**Tubal** The fertilized ovum implants in the fallopian tube, most frequently in the ampulla in humans (2, 3).	**Ovarian** The ovary is the site of implantation, growth, and development of the gestational sac (4).	**Cervical** The site of implantation is the cervical canal—a rare form of ectopic pregnancy in humans (5).	**Abdominal [peritoneal]** The fertilized ovum implants in the abdomen. The most common subtype of ectopic pregnancy recognized in animals other than non-human primates (1).	
			**Primary** Abdominal implantation is due to the fertilized ovum entering the peritoneal cavity, such as through the fimbria. Lack of lesions in the reproductive tract is suggestive of primary extrauterine pregnancy, but the possibility of repair cannot be discounted (6).	**Secondary** Uterine or tubal trauma leads to oocyte release into the abdomen (7).

Abdominal pregnancies have been previously described in laboratory (8), pet (9–11), and farmed *Oryctolagus cuniculus* (1, 12, 13). Indeed, in farmed rabbits, use of artificial insemination has been postulated to be a factor in the pathogenesis of some cases (12). Abdominal pregnancy in wild lagomorphs is likely under-reported, although two cottontail rabbits (*Sylvilagus floridanus*) (14), two European hares (*Lepus europaeus*) (15), and a mountain hare (*Lepus timidus scoticus*) (16) are amongst the reports of abdominal pregnancies in this group of animals. The present report describes a case of secondary abdominal pregnancy in a wild European rabbit, *O. cuniculus*, with concurrent impaired mammary gland development.

## Case Presentation

**Figure 1:**
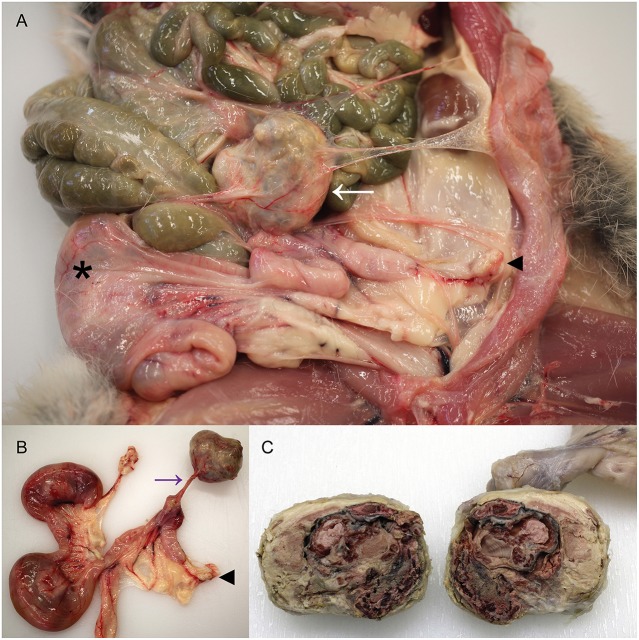
Presence of a lithopedion in a wild European rabbit**. (A)** A firm mass with a smooth surface is present in the abdomen of a female rabbit (white arrow). Asterisk denotes fetus in right uterine horn; arrowhead indicates left ovary. **(B)** A thin fibrovascular band (purple arrow) connects the lithopedion to the left uterine horn, which is distorted as a result. Arrowhead indicates left ovary. **(C)** On cut surface the lithopedion contains fetal remnants including skin, hair, bone, and hard palate (partially fixed tissue).

The cadaver of a wild female European rabbit, *O. cuniculus*, was one of a group shot for population management and donated for research and teaching to the veterinary anatomic pathology service of the Department of Veterinary Medicine, University of Cambridge (UK). The rabbit weighed 1.78 kg. Post mortem examination revealed a very firm, irregular mass with a smooth, gray/pink surface in the left abdomen. The mass had average dimensions 40 × 35 × 30 mm and had thin, tough, translucent adhesions to the left abdominal wall and the left uterine horn, the latter adhesion exhibiting evidence of vascularity, and the tension of the adhesion causing distortion of the left uterine horn (Figures 1A,B). On cut section, the mass was dry and multifocally gritty, but contained clearly discernable fetal remnants, including skin, hair, hard palate, and bone surrounded by lamellated fibrous tissue and fetal membranes (Figure 1C). The mass was interpreted to be a lithopedion, or mineralized abdominal ectopic pregnancy (17).

At the point of attachment to the left uterine horn, the uterine lumen contained an irregular, cream, 12 × 8 × 5 mm concretion and a circumferential 10 mm band of the uterine endometrium was dark red at this focus. The right uterine horn contained two fetuses, with crown-rump lengths 55 and 53 mm. Macroscopically, the mammary gland exhibited some evidence of development consistent with early pregnancy, such as prominent vasculature and mild parenchymal development, but this was considered markedly less than expected for this stage of pregnancy based on previous analyses (18). The gastrointestinal tract, in particular the stomach, was subjectively less full than the majority of wild rabbits from the same region examined as part of ongoing studies (18, 19) and scarce fecal pellets were present in the rectum. No other abnormalities were detected macroscopically.

Tissues were fixed in 10% neutral buffered formalin, and histological sections were prepared following standard procedures. The lithopedion was composed of amorphous eosinophilic material, indicative of degeneration and autolysis, which was multifocally heavily mineralized. Although nuclei were multifocally apparent, the degree of degeneration was such that cell populations were not identifiable and the presence of an inflammatory infiltrate could not be assessed. The uterus at the point of attachment of the lithopedion exhibited a locally extensive endometrial infiltrate of moderate numbers of lymphocytes, plasma cells, heterophils, and fewer macrophages, with locally extensive ulceration. Underlying this focus, there was a loss of distinction of the myometrial layers and locally extensive, mild to moderate, fibrosis. Together with the presence of the small concretion of mineralized material within the uterine lumen, this was considered strong evidence of abdominal pregnancy and formation of a lithopedion secondary to uterine rupture.

**Figure 2:**
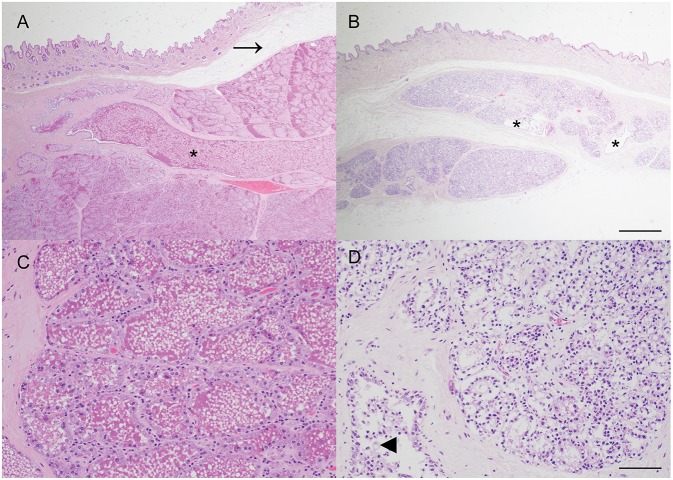
Impaired postnatal mammary development, consistent with physiologic agalactia, in a wild European rabbit with an abdominal pregnancy. **(A,C)** Mammary tissue from a doe with fetuses of a similar gestational age to the affected doe exhibits extensive alveolar development, and large amounts of eosinophilic proteinaceous material within mammary alveoli and ducts (asterisk). Arrow indicates artifactual separation of skin from underlying mammary tissue. **(B,D)** Mammary tissue from a female rabbit with an abdominal pregnancy exhibits alveoli with reduced diameter and lacking secretion, and small, largely empty ducts (asterisks). Some ducts contain small clusters of macrophages (arrowhead). Haematoxylin and eosin stain. Scale bars indicate 800 microns **(A,B)** and 80 microns **(C,D)**.

The macroscopic impression that the mammary gland was markedly under-developed for the stage of pregnancy was confirmed histologically, with only a thin layer of mammary tissue present and mammary lobules interspersed by prominent, wide, bands of connective tissue. The mammary alveoli frequently lacked intraluminal proteinaceous material and ducts were generally empty except for small numbers of macrophages and desquamated epithelial cells. The lack of mammary development was particularly pronounced when compared with mammary tissue from another female carrying a litter of similarly developed fetuses (crown-rump length median: 51 cm; mean: 50 cm; *n* = 8; Figure 2).

Histologically the lungs exhibited multifocal, mild, granulomatous and heterophilic pneumonia with multinucleated giant cells and very rare adiaspores, with morphology consistent with *Emmonsia* spp. There were no other significant findings on histological examination.

## Discussion

This report describes a case of secondary abdominal pregnancy in a wild rabbit. Secondary abdominal pregnancy is considered to arise as a consequence of traumatic injury to the reproductive tract, leading to a communication between the uterus or vagina and the peritoneal cavity, and the subsequent propulsion of a conceptus or fetus into the abdomen, potentially as a result of contractions associated with parturition in some cases (7, 12). In this case, the lithopedion was a similar size to the fetuses in the unaffected right uterine horn. It is therefore tempting to speculate that, given the degree of fetal maceration, the abdominal pregnancy originally arose during a previous pregnancy. This scenario is also consistent with the lack of detectable communication between the left uterine horn and the peritoneal cavity, and the presence of chronic inflammation and fibrosis in the myometrium suggestive of a chronic reparative process. The mineralized concretion within the uterus at this site likely represented residual material associated with the fetus expelled into the abdomen.

Studies in farmed or laboratory rabbits, for which dates of insemination are recorded, have facilitated accurate description of concurrent abdominal pregnancies and intrauterine fetuses of differing ages (13), suggesting that it is indeed feasible that the abdominal fetus described in this report originally arose during a previous pregnancy. This raises the question of whether the wild rabbit described here would have exhibited clinical signs associated with the presence of the lithopedion. Interestingly, during post mortem examination, it was felt that the stomach was less full than usual for rabbits examined as part of a cohort culled for population management, and the rectum was largely devoid of feces. It is therefore tentatively suggested that the lithopedion and associated adhesions were impacting the rabbit to a degree, supporting previous assertions that abdominal pregnancy may be associated with clinical signs in some cases: one pet rabbit diagnosed with an abdominal pregnancy was presented with inappetance and lethargy and subsequently died. Three mummified abdominal fetuses were detected during a post mortem examination, and although no definitive association with the death of the rabbit was described, no alternative cause of death was discovered (9). A further laboratory rabbit later discovered to have multiple abdominal lithopedions also exhibited anorexia (8). However, in another case in a pet rabbit, no clinical signs were noted (10).

Although no definitive relationship to the abdominal pregnancy can be inferred from this case, it is tempting to speculate on a potential relationship between the presence of the lithopedion and the impaired mammary development. Agalactia has been previously described in a laboratory rabbit with an extrauterine pregnancy although no description of the mammary gland was provided (8). In the present case, the relative lack of mammary development, when compared to an unaffected doe pregnant with similarly sized fetuses, is striking (Figure 2). One limitation of this case is that as the rabbit was wild, no reproductive history or details regarding concurrent lactation are available. The lack of postnatal mammary development, consistent with physiologic agalactia, may be attributable to a postulated lack of concurrent lactation due to loss of the previous litter, or the presence of just two viable fetuses, but it also seems possible that stress, resulting from the abdominal pregnancy and leading to elevated blood cortisol, may have played a more direct role. Secretion of prolactin, a key regulator of development of the milk secreting mammary epithelial cells via activation of Signal Transducer and Activator of Transcription 5 (STAT5) (20), is reduced in a rabbit model of critical illness using deep dermal burns (21). The impact of other stressors, such as heat stress, on postnatal mammary gland development are widely recognized in production ruminants (22). In this case there was no evidence of inflammation within the mammary gland and thus an infectious cause of agalactia was considered unlikely. This case highlights that further investigation of a potential link between abdominal pregnancy and impaired mammary gland development in rabbits may be merited.

The rabbit in this report was microscopically diagnosed with mild granulomatous and heterophilic pneumonia with multinucleated giant cells and very rare adiaspores, with morphology consistent with *Emmonsia* spp. We have recently described adiaspiromycosis in a wild European rabbit with a much higher burden of adiaspores and marked granulomatous pneumonia which was grossly apparent (19). By comparison, in this case the pathology was subtle, only appreciable histologically, and was considered to be most likely an incidental finding of no clinical consequence. Such incidental diagnosis of adiaspiromycosis has also been reported in other small mammal species such as moles (*Talpa europaea*) (23). DNA extraction and subsequent PCR amplification and sequencing would have been the optimal approach to attempt to definitively confirm the diagnosis of adiaspiromycosis in this rabbit, particularly as culture is oftentimes unrewarding (24).

This case report illustrates that, although abdominal pregnancies in rabbits may be associated with artificial insemination procedures in some farmed rabbits (12), they may also occur in wild *O. cuniculus*. This report also suggests that an abdominal pregnancy should be considered as a differential diagnosis in the investigation of a palpable abdominal mass or masses in *O. cuniculus*. Larger scale studies examining ectopic pregnancy in rabbits may reveal further insights into the pathogenesis of this condition.

## Data Availability

All datasets analyzed for this study are included in the manuscript.

## Ethics Statement

The animal study was reviewed and approved by University of Cambridge Department of Veterinary Medicine Ethics and Welfare Committee.

## Author Contributions

KH performed the gross and histological post mortem examination, conceived the study, collected and collated the data, and wrote the manuscript.

### Conflict of Interest Statement

The author declares that the research was conducted in the absence of any commercial or financial relationships that could be construed as a potential conflict of interest.
